# Occupational health risks of pathologists - results from a nationwide online questionnaire in Switzerland

**DOI:** 10.1186/1471-2458-12-1054

**Published:** 2012-12-06

**Authors:** Florian Rudolf Fritzsche, Constanze Ramach, Davide Soldini, Rosmarie Caduff, Marianne Tinguely, Estelle Cassoly, Holger Moch, Antony Stewart

**Affiliations:** 1 Faculty of Health, Staffordshire University, Stoke on Trent, United Kingdom; 2 Institute of Surgical Pathology, University Hospital Zurich, Zurich, CH-8091, Switzerland; 3 Institute of Pathology, Cantonal Hospital St. Gallen, St. Gallen, Switzerland

**Keywords:** Occupational, Health risk, Pathologist, Musculoskeletal, Injury, Questionnaire

## Abstract

**Background:**

Pathologists are highly trained medical professionals who play an essential part in the diagnosis and therapy planning of malignancies and inflammatory diseases. Their work is associated with potential health hazards including injuries involving infectious human tissue, chemicals which are assumed to be carcinogenic or long periods of microscope and computer work. This study aimed to provide the first comprehensive assessment of the health situation of pathologists in Switzerland.

**Methods:**

Pathologists in Switzerland were contacted via the Swiss Society of Pathologists and asked to answer an ethically approved, online anonymous questionnaire comprising 48 questions on occupational health problems, workplace characteristics and health behaviour.

**Results:**

163 pathologists participated in the study. Forty percent of pathologists reported musculoskeletal problems in the previous month. The overall prevalence was 76%. Almost 90% of pathologists had visual refraction errors, mainly myopia. 83% of pathologists had experienced occupational injuries, mostly cutting injuries, in their professional career; more than one fifth of participants reported cutting injuries in the last year. However, long lasting injuries and infectious diseases were rare. Depression and burnout affected every eighth pathologist. The prevalence of smoking was substantially below that of the general Swiss population.

**Conclusions:**

The results of this study suggest that more care should be taken in technical and personal protective measures, ergonomic workplace optimisation and reduction of work overload and work inefficiencies. Despite the described health risks, Swiss pathologists were optimistic about their future and their working situation. The high rate of ametropia and psychological problems warrants further study.

## Background

The UK Royal College of Pathologists describes pathology as a hidden science that helps doctors to make decisions and save lives [1]. Pathology is sometimes regarded as a “hidden science”, arising from a public misconception that the work of pathologists exclusively involves performing autopsies, which misses the more important tasks of diagnosing and characterising tumours and infectious diseases in living patients. Numerous potential health hazards are associated with the routine work of pathologists, but publications on this topic are rare and often decades old [2-11]. While pathologists might feel hidden and neglected by the general public, they themselves may neglect the health risks associated with their own work.

Harrington and colleagues analysed causes of death of pathologists in the United Kingdom from the 1950s until the late 1980s [4-6]. They found excess death rates due to suicide and also higher rates of brain tumours and haematopoietic and lymphatic malignancies. Among the hypothetical causes of these findings was exposure to formaldehyde, a toxic, irritant and possibly carcinogenic substance. Formaldehyde is widely used in a watery solution called formalin in pathology laboratories worldwide for the fixation of human tissues [10,12-14]. Data on the association between formaldehyde exposure and cancer are not consistent, however reported malignancies include brain tumours, lympho-haematopoietic neoplasms, nasopharyngeal and pancreatic cancers [13,15-19]. Exposure to formaldehyde can also cause allergies and irritative reactions, often acquired by an occupational exposure to formaldehyde releasers like liquid soaps or detergents [20].

Biohazards are another intrinsic health risk for pathologists due to their work with potentially infective fresh human tissues during organ dissection, operative frozen section and autopsy [21,22]. While there is risk of human immunodeficiency virus (HIV) and hepatitis C (HCV) transmission, reported cases are very rare [23-25]. Working in close proximity to infectious aerosols, contact with tubercle bacilli, needle puncture and cutting injuries are other possible sources of infection [11,26-28].

Personal protective gear, including vision shields, protective glasses, particulate filter respirators and thin Kevlar enhanced cut-resistant protective gloves are available and highly effective. Kevlar enhanced gloves have been shown to effectively prevent cutting injuries, but compliance in wearing these gloves is relatively poor [9,29-32].

A publication by George [3] and the related reply [33] elucidate another well known but often neglected health problem in pathology laboratories[3,33]. The daily routine work of pathologists includes several hours of microscope and computer work [3]. Microscopes are often not ergonomically optimized and do not allow a neutral sitting position, requiring the pathologist to bend forward [3,8,34]. A survey among 244 cytotechnologists, a typical example of microscope-using employees, demonstrated that more than 80% of participants suffered from musculoskeletal discomfort including headache, neck pain, stiffness, back pain and upper-extremity discomfort [35]. Interestingly, less than one third of cytotechnologists reported having an ergonomic assessment of their workplace. Thompson et al. concluded that basic ergonomic training and use of ergonomic aids could substantially reduce musculoskeletal discomfort [35]. Unfortunately recommendations for such workplaces by the Centers for Disease Control and Prevention (CDC), modified by George, do not appear to match real life working situations [3]. In 2010 Flavin et al. reported similar symptoms and results including eye fatigue from microscopic work in an Irish cohort of pathologists and cytologists [2].

The problem of workplace-related musculoskeletal problems of pathologists is not new [3]. Publications from Finland and Austria have provided images of sitting postures at microscopes from 2002, 2003 and 2010 as well as potential solutions for corresponding musculoskeletal problems that illustrate these issues [3,8,34]. The additional suggested positive effects of ergonomic principles and ergonomic interventions at workplaces include improved morale and productivity [36,37].

In this study, occupational health risks of pathologists in a multilingual European country are systematically analysed and subsequently discussed in relation to the literature.

## Methods

### Study design

This study was based on an anonymous online questionnaire comprising 48 questions. Separate versions of the questionnaire were available for the three main national languages of Switzerland: German, French and Italian (see Additional file 1). The online survey resource Q-set.de (http://en.q-set.de/, Goldecker LLC, Orhalm, Germany) was used as an online platform for the questionnaires. Access to the questionnaires was only possible after completion of an informed consent form. The informed consent form was electronically submitted and could not be connected to a corresponding questionnaire. The study was approved by the cantonal ethical committee Zurich (KEK-ZH-Nr. 2011-0054/0) and by the ethical committee at Staffordshire University.

### Participant selection

The study aimed to include all pathologists currently working in Switzerland, including residents in pathology. Potential participants were approached by email, via the Swiss Society of Pathologists (SSP, http://www.sgpath.ch/infos.html). The SSP sent short information emails with the links to the consent form for all three variants of the questionnaire to members of the SSP and to all other pathologists in Switzerland for whom the SSP held contact data. For larger institutes of pathology, the email was sent to head physicians with the request to send it also to their medical employees. After 6 weeks, a reminder mail was send by the SSP. This procedure was intended to reach more than 90% of pathologists and residents in pathology in Switzerland. The total number of these was estimated to be between 250 and 300 (personal unpublished communications with the secretary of the SSP).

Participation was rewarded with optional participation in a raffle to win a Scandinavian designed ergonomically optimized office chair (Capisco Puls, HÅG Scandinavian Business Seating AS, Oslo, Norway).

### Rationale

The chosen methodology relies on four main assumptions. First, a study requiring the physical attendance or visits to personnel was not practical. Second, computer and internet access were essential prerequisites for the work of pathologists. The SSP provided contact data and coverage of Swiss pathologists was thereby maximised. Third, unlike interviews, the online questionnaire provided anonymity while ensuring informed consent. Fourth, the chosen methodology was cost-effective; the raffle and the official SSP support were motivating factors for participation.

### Statistical analyses and data screening

Statistical analysis was performed using IBM SPSS Statistics 19.0 (IBM, Armonk, NY, USA). In terms of sample size calculation, the target sample size was the whole community of Swiss pathologists. The final sample size of 163 pathologists was above the suggested sample size for an estimated whole population of 250 or 275 pathologists but just below that for an estimated population of 300 pathologists.

Kolmogorov-Smirnov tests indicated a non-normal distribution. Transformation did not normalise the data, so nonparametric techniques were used. Missing value analyses showed that with the exception of two open questions (“reasons for the personal estimation on the future relevance of pathology” and “general comments on the questionnaire”), missing values were below 10% and with the exception of 6 parameters below 5%. Descriptive statistics, frequency analyses, Chi-square-tests, Fisher’s-exact-tests, logistic regression and bivariate correlations (Spearman) were applied.

## Results

### Participation

163 pathologists answered the questionnaire. This corresponds to an uptake of 54% - 65% by the 250–300 pathologists in Switzerland. Further details on cohort characteristics are summarized in Table 1. The German, French and Italian version of the questionnaire was used by 71.8%, 21.5% and 6.7% of participants respectively. Since many pathologists in Switzerland are multilingual, the language chosen did not represent any specific part of the country.

**Table 1:** General cohort characteristics of pathologists in Switzerland

**Parameter**	**Number (%)**	**Parameter**	**Number (%)**
Gender		Current position	
Women	89 (54.6%)	Resident	41 (25.2%)
Men	73 (44.8%)	Consultant	121 (74.2%)
Age		Working atmosphere	
25-35 years	45 (27.6%)	Very good	60 (37.3%)
36-45 years	56 (34.4%)	Good	75 (46.6%)
46-55 years	43 (26.4%)	Medium	21 (13.0%)
>55 years	19 (11.7%)	Bad	2 (1.2%)
Place of work		Mean weekly working hours	
Private practice	29 (17.8%)	≤50h	68 (41.7%)
University hospital	65 (39.9%)	>50h	65 (39.9%)
Non-University hospital	68 (41.7%)	>60h	26 (16.0%)
Part time work		Part time work specification	
No	119 (73.0%)	(n=43)	
Yes - ≥80%	25 (15.3%)	Part time work only short term	10 (23.3%)
Yes - ≥60%	9 (5.5%)	Part time work > 3 years (long term)	25 (58.1%)
Yes < 60%)	9 (5.5%)		
Work manageable in working time		Work predominantly efficiently organised	
Yes	84 (51.5%)	Yes	104 (65.0%)
No	71 (44.8%)	No	51 (31.3%)
Regular sports		Estimated future relevance of pathology	
No	43 (26.4%)	Increasing	
Yes >1x/week	68 (41.7%)	Remaining the same	96 (58.9%)
Yes 1x/week	33 (20.2%)	Decreasing	49 (30.1%)
Yes >1x/month	13 (8.1%)		17 (10.4%)
Yes <1x/month	5 (3.1%)		
Personal 2-year perspective		Personal 5-year perspective	
Very good	51 (31.3%)	Very good	44 (27.0%)
Good	86 (52.8%)	Good	90 (55.2%)
Rather bad	15 (9.2%)	Rather bad	15 (9.2%)
bad	1 (0.6%)	Bad	2 (1.2%)

### Health conditions

Table 2 summarizes results on the reported health problems of pathologists in Switzerland.

**Table 2:** Health status parameters of pathologists in Switzerland

**Characteristics**	**Yes (%)**	**No (%)**
Ever received an introduction into work place ergonomics	24 (14.7%)	136 (83.4%)
Ever experienced work-related musculoskeletal problems	123 (75.5%)	40 (24.5%)
Musculoskeletal problems in the last four weeks (n=123)	69 (56.1%)	53 (43.1%)
Signed off due musculoskeletal problems last year (n=123)	11 (8.9%)	112 (91.1%)
Doing regularly short breaks for stretching exercises	36 (22.1%)	124 (76.1%)
Any known ametropia	145 (89.0%)	17 (10.4%)
Aggravation of ametropia since working in pathology (n=145)	82 (56.6%)	58 (40.0%)
Eye fatigue symptoms in the last month	90 (55.2%)	68 (41.7%)
Ever experienced occupational injuries in pathology	135 (82.8%)	28 (17.2%)
Experienced such injuries in the last year (n=135)	37 (27.4%)	95 (70.4%)
Remaining permanent damages from such injuries (n=135)	5 (3.7%)	127 (94.1%)
Ever experienced intolerance reactions against formalin	41 (25.2%)	118 (72.4%)
Any known allergy	55 (33.7%)	105 (64.4%)
Since working in pathology ever diseased with…		
Tuberculosis	2 (1.2%)	153 (93.9%)
Positive tuberculin test only	31 (19.0%)	119 (73.0%)
Hepatitis B	1 (0.6%)	154 (94.5%)
Hepatitis C	0 (0.0%)	155 (95.1%)
HIV	0 (0.0%)	154 (94.5%)
Burnout	14 (8.6%)	141 (86.5%)
Depression	11 (6.7%)	144 (88.3%)
Hypertension	11 (6.7%)	144 (88.3%)
Diabetes mellitus type II	3 (1.8%)	152 (93.2%)
Malignancies	3 (1.8%)	149 (91.4%)
Sufficient hepatitis B immunisation	153 (93.9%)	9 (5.5%)
Ever received BCG (tuberculosis) immunisation	128 (78.5%)	31 (19.0%)
Smoking	17 (10.4%)	145 (89.0%)
Cut-resistant gloves available at workplace	99 (60.7%)	60 (36.8%)
Regular personal use of cut-resistant gloves at dissections/autopsies	39 (23.9%)	108 (66.3%)
Extraction of air in the dissection room considered sufficient	94 (57.7%)	66 (40.5%)

Percentages relate to the whole cohort of 163 participants unless stated otherwise. Respective sub-set numbers are then provided in the characteristics column.

#### Musculoskeletal problems

Musculoskeletal problems affected more than three quarters of Swiss pathologists with more than 40% of pathologists having suffered from these problems during the past four weeks. Seven percent of pathologists had been on sick leave due to musculoskeletal problems during the last year for a median of 4 (mean = 6) days. For the overall prevalence of musculoskeletal problems, logistic regression analysis suggested a higher risk for female pathologists (p=0.024, effect coefficient (EC): 0.282), more weekly working hours (p=0.023, EC: 2.489) and surprisingly, fewer hours spent at the computer (p=0.033, EC: 0.801). Regression analysis for the one-month prevalence of musculoskeletal problems did not indicate any significant explanatory factor. Impossibility of a straight line of sight (microscopes with extensive adjustability of tubes and oculars to obtain a straight line of sight through the oculars of the microscope without the need to bend forward) was the factor closest to reaching significance (p=0.097, EC: 2.235).

**Figure 1:**
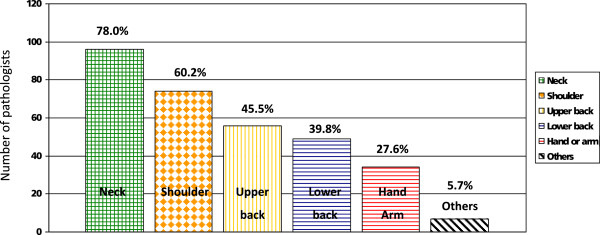
**Location of musculoskeletal problems in Swiss pathologists.** The bars reflect the number of pathologists complaining about pain in the respective location. Percentages relate to the 123 pathologists suffering from musculoskeletal problems.

The most common locations for musculoskeletal problems were neck and shoulders (Figure 1). Other locations and problems included hip, face/head, ankles and a so called “pathologists’ hump” (allegedly related to microscope work).

**Figure 2:**
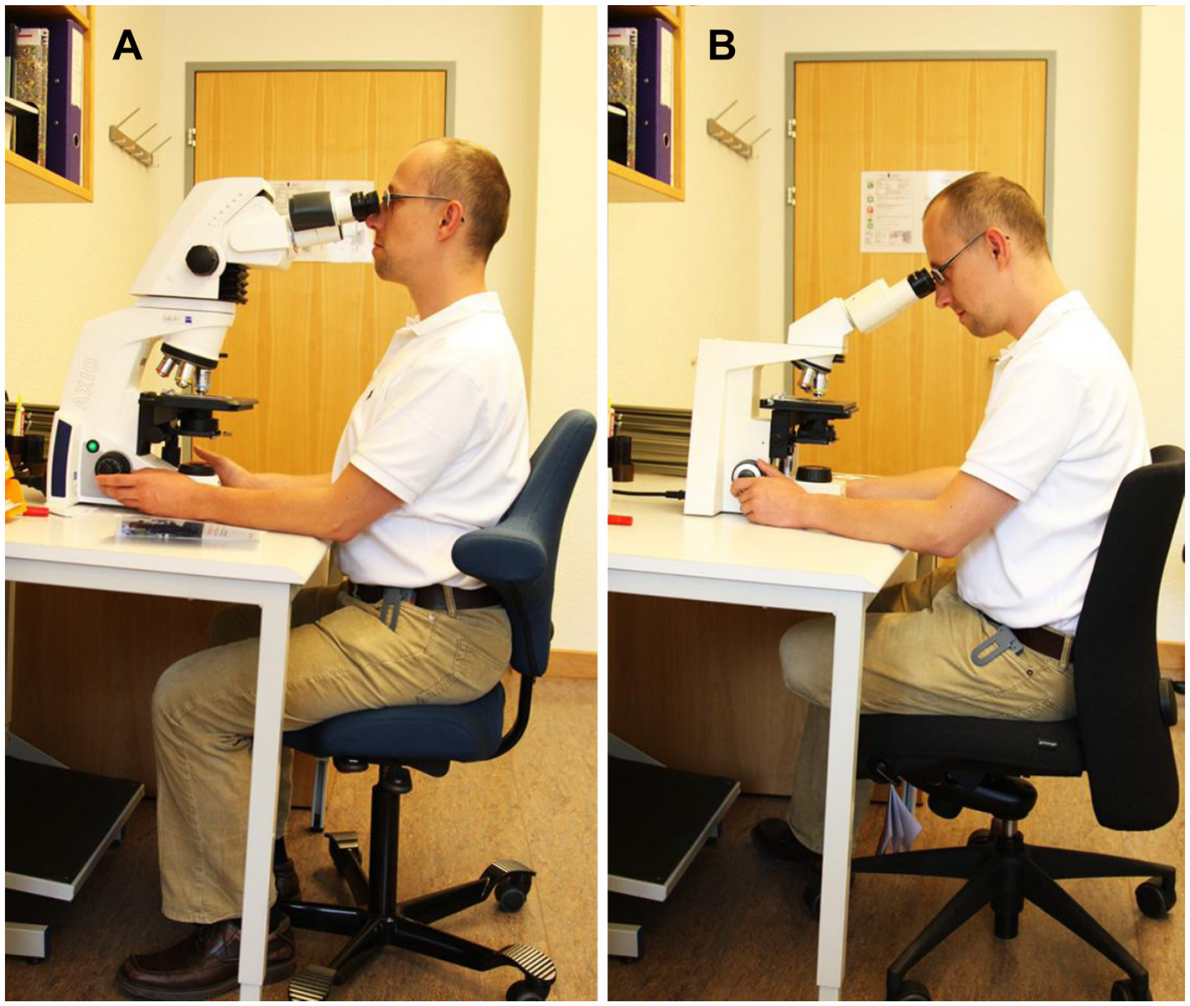
**Ergonomically optimized and conventional microscopes and office chairs.** Comparison of seating position and neutral relaxing posture between ergonomically optimized office chair (HÅG) and microscope (Zeiss) (**A**) and a conventional office chair and microscope (**B**).

**Figure 3:**
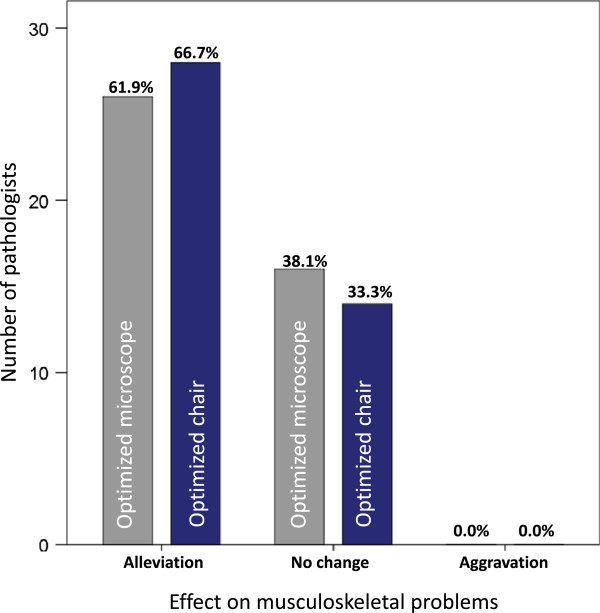
**Effect of ergonomically optimized equipment on musculoskeletal problems.** The bars reflect the number of pathologists who commented on the effect of an ergonomically optimized microscope (grey) or office chair (blue) on their musculoskeletal problems. Percentages relate to the 42 pathologists who answered this question.

The prevalence overall, or in the last month, of musculoskeletal problems was no lower among those with ergonomically optimized chairs (Figure 2) than among those without them. However, the majority of pathologists who used ergonomically optimized office chairs felt their symptoms were alleviated by the chairs (Figure 3). More than 60% of pathologists reported alleviation of their musculoskeletal problems by the use of an ergonomically optimized microscope (Figure 2 &3). There were no statistically significant differences between different microscope manufacturers. Neither the habit of regular breaks for stretching exercises, hours spent working at the microscope or at the computer, work experience, adjustability of the microscope tube or ability of horizontal line of sight, desk characteristics, participation in sport nor presence or absence of workplace ergonomics introductions were significantly associated with the overall or one-month prevalence of musculoskeletal problems.

#### Visual problems

**Figure 4:**
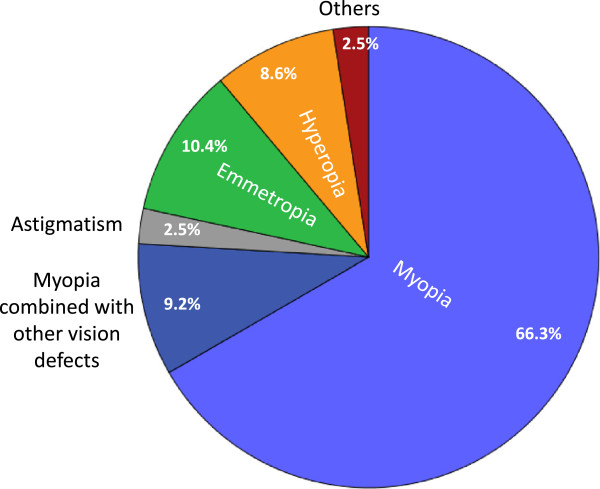
**Distribution of different visual impairments among Swiss pathologists.** The pie chart depicts the distribution of visual impairments and emmetropia among Swiss pathologists. Percentages relate to all 163 pathologists participating in the study.

The prevalence of ametropia, defined as any kind of refractive error of the eyes, was very high in this occupational group (90%). Myopia was the most common type of ametropia affecting about 75% of pathologists and constituting almost 85% of all vision defects (Figure 4). More than 80% of the affected pathologists had a visual impairment already before they have started working in pathology. More than 50% of ametropic pathologists had experienced an aggravation of ametropia during their work in pathology with a median aggravation of 1.5 dioptres.

#### Injuries

**Figure 5:**
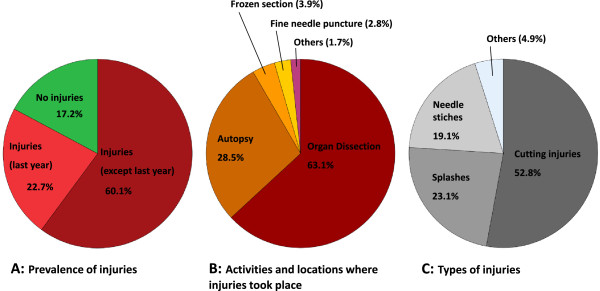
**Injury characteristics of pathologists in Switzerland.** The pie charts illustrate the prevalence of injuries among pathologists (**A**), the activities and locations where injuries took place (**B**) and the types of injury (**C**). Percentages relate to all 163 study participants (**A**), all 179 activities and locations given (**B**) and all 225 types of injury provided (**C**).

One-hundred and thirty-five pathologists (82.8%) reported at least one injury during their career in pathology. Injury characteristics are shown in Figure 5. One-hundred-twenty-five of these 135 participants (93%) reported activities and locations where injuries took place. The most common activities were macroscopic organ dissection and autopsy.

The most common types of injury were cutting injuries followed by splashes of fluids or organic materials to mucous membranes. Rare other types of injury included formalin contact to skin and mucous membranes, different types of stitches and special cutting injuries from cover glasses, knives or wires.

Pathology residents were much more often affected by injuries than consultants during the previous year (Fisher’s exact test: p=0.001). Interestingly pathology residents were more often using cut-resistant protective gloves (51% versus 18%) in comparison to consultants (Fisher’s exact tests: all p<0.001,). Concordantly the use of cut-resistant gloves was highest in university hospitals followed by non-university hospitals, being both typical training and teaching centres. The lowest values were reported from private practices (χ2=11.622, p=0.003,). For more than one third of pathologists in Switzerland such gloves were not available.

Surprisingly, the prevalence of injuries during the last year, including cutting injuries, did not differ significantly between reported users and non-users of these gloves. Nonetheless, glove-users career in comparison to non-users overall (Fisher’s exact test: p=0.007).

#### Formalin, allergies and other health problems

Intolerance reactions to formalin including severe mucosal or skin irritation, mucosal inflammation, fatigue or sleep disorders were experienced by one quarter of pathologists.

**Figure 6:**
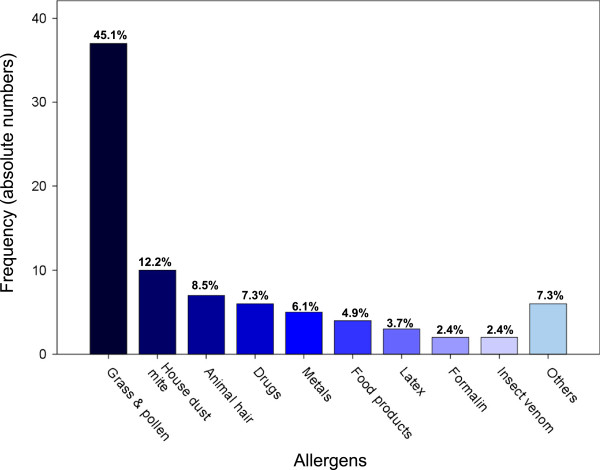
**Types of allergy of pathologists in Switzerland.** The bars reflect the numbers of allergens reported by pathologists suffering from allergies. Grass and pollen were the most common allergen. Percentages relate to the 82 mentioned allergens in this study.

In terms of allergies, 34.2% of pathologists were affected. The leading allergens were grass and pollen followed by house dust mites (Figure 6).

The most common mental and systemic diseases of pathologists were burnout, depression and arterial hypertension (Table 2). The former two were significantly associated with each other (Fisher’s exact test: p=0.001) and together affected almost every eighth pathologist (12.3%). Older pathologists were at higher risk for burnout or depression (Logistic regression: p=0.024, EC: 5.489). Arterial hypertension was more common in older pathologists (χ2= 12.102, p=0.007) but was not associated with smoking or participation in sport.

Smoking was generally rare among pathologists. Gender and age ratios for smoking were even.

Only three malignancies were reported in this cohort. They included two germ cell tumours and one bladder cancer.

Tuberculosis was also very rare in this cohort with just two pathologists affected. Interestingly almost every fifth pathologist had a positive tuberculin test during his/her career in pathology.

Other infectious diseases were exceptional (1 hepatitis B case) to non-existent in this cohort, and the immunisation rate for hepatitis B (HBV) was high (94%).

### Work and cohort characteristics

Over half of the pathologists (56%) reported working an average of more than 50 hours per week. More than half of those pathologists with ≤50 hours per week were employed on a part-time basis. Thirty-nine per cent of female and twelve per cent of male pathologists were part-time workers (Fisher’s exact test: p<0.001). More than 90% of participating pathologists used a microscope on a daily basis, and more than 95% worked daily on a computer. Median daily working hours were five and four hours for microscope and computer respectively. 35% and 26% of pathologists reported working more than 5 hours at the microscope and computer. Hours of daily microscope work were significantly correlated with work experience (Spearman bivariate correlation: p<0.001, correlation coefficient 0.441) and weakly correlated with dioptres worsening during work in pathology (Spearman bivariate correlation: p=0.045, correlation coefficient 0.235). More than five hours of computer work per day resulted in an adverse rating of the working atmosphere (Fisher’s exact test: p=0.039). Workplace characteristics are listed in Table 3.

**Table 3:** Workplace specifications and ergonomic equipment of pathologists in Switzerland

**Characteristics**	**Yes (%)**	**No (%)**
Office		
At least one window	159 (97.5%)	2 (1.2%)
Air conditioning	36 (22.1%)	122 (74.8%)
Shared with colleagues	54 (33.1%)	105 (64.4%)
Microscope		
Tube adjustable in height	95 (58.3%)	65 (39.9%)
Regularly serviced	130 (79.8%)	28 (17.2%)
Allows horizontal line of sight	99 (60.7%)	61 (37.4%)
Office chair		
Individually adjustable	149 (91.4%)	11 (6.7%)
Ergonomically optimised	66 (40.5%)	94 (57.7%)
Office desk		
Sufficiently adjustable in height	62 (38.0%)	99 (60.7%)
Big enough	118 (72.4%)	43 (26.4%)
Inclination adjustable	6 (3.7%)	155 (95.1%)

With a market share of more than 60% of study participants, Zeiss was the most frequently reported microscope manufacturer for pathologists in Switzerland, followed by Olympus, Nikon and Leica. About 90% of Nikon and Olympus microscopes had adjustable tubes, compared to less than 50% of those manufactured by Zeiss and Leica (χ2=24.693, p<0.001). Similar results were found for the allowance of a horizontal line of sight (χ2=14.034, p=0.007).

## Discussion

To our knowledge, this study represents the first general health assessment of pathologists. The feedback rate of up to 65% is higher than that of most previous studies [2,10,38].

The prevalence of musculoskeletal problems among pathologists was high. It is interesting to note that these problems were also experienced by many young pathologists. This argues against a mere aging effect of this disorder, and also underlines the importance of addressing this problem not only on middle aged to older employees but in the younger ones, at an early stage in their career. Musculoskeletal disorders are common in the general public and constitute one of the major causes of work absenteeism in developed countries [39-41]. Female gender, higher age and low socioeconomic status are associated factors [42]. The prevalence of upper extremity symptoms in working populations is estimated to be between 20%-30% [40]. While increased working hours were associated with musculoskeletal problems, other factors such as working time at the microscope/computer or ergonomic workplace settings were not; this is in agreement with a study by Lorusso et al. [43]. The lack of an association of ergonomic workplace settings with lower prevalence of musculoskeletal problems, at least in terms of the one-month prevalence, is surprising. A possible explanation could be that the ergonomic equipment may have been acquired secondary to musculoskeletal disorders. Thereby the negative selection of affected pathologists might have concealed any positive effects of the ergonomic devices. The finding that 62% to 67% of pathologists with musculoskeletal problems who switched from a conventional microscope or office chair to ergonomically optimized models reported a relief of their pain further supports this theory.

Visual refractive errors are more common in pathologists than in the general population, university students or other hospital workers [44-49]. It is possible that ametropic students may choose more likely to enter this discipline. On the other hand, the work of pathologists is associated with possibly eye-straining activities such as long lasting microscopy and computer work [50-53]. The aggravation of ametropia while working in pathology, experienced by 50% of participants, might be part of the normal time course of conventional myopia, yet it may also be associated with the continuous near-field work required [54].

The analysis of injuries among pathologists suggests that injuries with sharp and possibly infectious instruments (mainly knives and needles), are almost an integral part of a pathologists’ career. With about 83% of pathologists affected, these injuries constituted the most common harm in pathology. Cutting injuries were the predominant type of injury. The predominance of residents among the injured pathologists may be explained either by their lack of experience, the training situation or by the fact that, unlike consultants, residents are more at risk as they are typically deployed on a daily basis in macroscopic organ dissection or autopsies. The few pathologists who stated having never experienced an injury in their pathology career, were mainly experienced senior consultants. With 26% of the never-injured pathologists, the use of cut-resistant gloves in this group was twice as high as in those consultants who have had experienced injuries. Seven of the eight residents who reported no injury in their pathology career ≤1 year work experience. Only one of those residents reported not using cut-resistant gloves. However, given the perspective of around 4–5 years of further training, the chance for future injuries during the course of their residency should not be neglected. Recognising that the use of cut-resistant gloves was rare among elder pathologists and assuming that dexterity was not substantially different between generations of pathologists, the possibility of recall bias must taken into account when interpreting the 19 never-injured consultants. One of the elder pathologists rightly commented that some of the retrospective data should be taken “cum grano salis” (Latin: with a pinch of salt).

Cut-resistant gloves, worn between conventional vinyl gloves, can protect users from cutting injuries [29]. Loss of sensitivity and dexterity are reasons for rejection of this protective measure [29-32,55]. Splashes to mucous membranes, constituting the second most common type of injury, should be preventable by protective glasses or facemasks. This study did not analyse the availability or the use of such masks.

A total of 94% of those pathologists injured reported no lasting damage from these events. This finding might support the somewhat fatalistic behaviour of many consultants when rejecting cut-resistant gloves and accepting cutting injuries as an inconvenient but harmless requirement in pathology. Reports on infections resulting from such injuries are also very rare [21,23,24]. However the possibility of serious consequences exists, and the reduction of injuries in these high-risk fields should be given high importance.

While HBV vaccination of medical professionals is a standard precautionary measure in most developed countries, a recent study from Lithuania revealed an alarming rate of almost 90% of non-vaccinated medical staff members [56]. Meanwhile hepatitis B immunisation levels among Swiss pathologists were high. Nonetheless, about 5% of pathologists, almost exclusively senior consultants, reported being insufficiently immunized.

Formalin is the most widely used fixative in Swiss pathology institutes (unpublished personal experience). The effects of formaldehyde on human health are not well understood, especially in terms of malignancies [13,15,57,58]. Malignancies were very rare in Swiss pathologists. No brain tumours or lympho-haematopoietic malignancies – previously believed to be pathology-associated malignancies - were reported [5]. Although these results argue against major carcinogenic effects of formaldehyde exposure in a pathology setting, some caution is warranted due to possible recall bias, selection bias and small numbers [59,60].

Intolerance reactions to formalin were reported by 25% of pathologists but specific allergies against formalin as well as against latex are rare [10,14]. Whether a complete abandonment of formaldehyde in pathology is reasonable and feasible will depend on further proof of adverse effects of formaldehyde, safety and economic feasibility of alternative agents and requirements of a future more molecular-based spectrum of analyses.

Smoking prevalence in Switzerland has been decreasing slowly for several years and was around 30% in 2010, with more men smokers than women [61]. In 2007 and 2009 12% to 17% of Swiss primary care physicians were active smokers [62,63]. The prevalence in Swiss pathologists is even lower (10%). Along with a possible trend for the desired response, the daily confrontation with fatal consequences of smoking in the morgue and in cancer diagnostics might present one possible explanation. Furthermore the smoking ban in several Swiss hospitals, bars and restaurants may also have promoted lower smoking rates.

Interestingly, burnout and depression were not rare among study participants. The burnout prevalence in other medical professions ranged from 4% to 40% depending on the degree of burnout [64-67]. In these studies high workload, more than 50 working hours per week and frequent interruptions were factors associated with burnout.

The association between work efficiency and burnout and as a trend with depression can be interpreted differently. While insufficiently organised work can be depressing, people suffering from burnout or depression might rate their surroundings, perspectives and also work efficiency as being even less satisfactory. Nonetheless, employee-centred workflow optimisation should be taken seriously, especially since almost half of pathologists were unable to finish their work within regular working hours. Nearly one third of pathologists considered the workflow in their institution to be inefficiently organised.

Tuberculosis is often considered a ‘pathologists’ disease’ and has been demonstrated to affect pathologists much more often than the general population and other professional medical groups [26,28,68,69]. In comparison, the prevalence of a history of tuberculosis in our cohort was low (1.2%). The tuberculosis skin test is considered a good test for tuberculosis control. It is also interesting that in comparison to other Swiss study cohorts a high percentage of pathologists reported having a positive tuberculin skin test during their time in pathology [70-72]. Almost 80% of pathologists had a BCG vaccination during their lifetime. Six to 10% of positive skin tests are thought to be attributable to a previous BCG vaccination but after more than 10 years after the vaccination it should no longer be considered in the interpretation of a positive test result [73,74]. According to these data, the vaccination could explain a positive skin test in 8–10 pathologists within this study. That the number is three times as high might reflect a high level of infection with tubercle bacilli, which might remain in the body in an inactive state. Therefore the notion that pathologists are at increased risk of tuberculosis infection cannot be ignored. This underlines the importance of routine precautionary measures such as effective respirator masks.

Another important finding of this study is that pathologists are generally very positive about their working atmosphere, personal work-related future perspectives and the future relevance of pathology as a medical discipline. Reasons for a positive rating of the future relevance of pathology included the relevance of pathology to medicine and especially for oncologic therapy planning, the individualisation of therapies with the need for very specific pathologic diagnoses and the future importance of molecular pathology to answer prognostic and predictive questions. Reasons for a decreasing relevance of pathology included performing fewer autopsies, the reduction of pathology in the curricula of medical students, the introduction of the DRG (diagnosis related groups) system in Switzerland and the fear that attractive diagnostic tests might be taken over by other medical disciplines.

## Conclusions

This online questionnaire study is the first comprehensive occupational health assessment of a nationwide cohort of pathologists. Most pathologists in Switzerland are optimistic, long-working, physically active, normotensive non-smokers who are comfortable with their current working situation. The most common health problems include cutting injuries, ametropia, eye fatigue symptoms and musculoskeletal disorders. Formaldehyde intolerance symptoms, burnout and depression are also common. In terms of preventive actions, effective personal protective measures to reduce injuries, a further reduction in formaldehyde exposure, ergonomic improvements of pathologists’ workplaces and evaluation of work processes to improve efficiency are recommended.

### Consent

Written informed consent was obtained from the person pictured in Figure 2 for publication of these images. A copy of the written consent is available for review by the Series Editor of this journal.

## Abbreviations

BCG: Bacille Calmette-Guerin; CDC: Centers for Disease Control; DRG: Diagnosis Related Groups; EC: Effect coefficient; HIV: Human immunodeficiency virus; HBV: Hepatitis B virus; HCV: Hepatitis C virus; SSP: Swiss Society of Pathology.

## Competing interests

The authors declare that they have no competing interests.

## Authors’ contributions

FRF conceived and conducted the study and wrote the manuscript. CR produced the French version of the questionnaire and supported its evaluation. DS created the Italian version of the questionnaire and supported its evaluation. RC and HM supervised the study from the pathological and administrative perspective. EC and MT supported the production of the French questionnaire and assisted in the application to the ethical committee in Zurich. AS was University supervisor for the study, provided support in terms of content and logistics, statistical analysis and manuscript conception. All authors read and approved the manuscript.

## Pre-publication history

The pre-publication history for this paper can be accessed here:

http://www.biomedcentral.com/1471-2458/12/1054/prepub

## Supplementary Material

Additional file 1 Questionnaire: English version of the questionnaire. Click here for file
