# Supplementary material for: Occupational health risks of pathologists - results from a nationwide online questionnaire in Switzerland
Source: BMC Public Health. 2012 Dec 6;12:1054. doi: 10.1186/1471-2458-12-1054 (PMC3538703; doi:10.1186/1471-2458-12-1054)
Supplement: Additional file 1 — Questionnaire: English version of the questionnaire. [file 1471-2458-12-1054-S1.doc]

**English print version of the questionnaire**

# Information, consent form and raffle

Dear pathologist,

Thank you very much for following this link. We would like to ask you for your consent to a questionnaire of a study on health aspects and health risks in pathology.

The background of this study: Until now no or only limited data on pressure of work, health risks and their consequences for pathologists is known. However, work in pathology has been associated with several specific health risks. This questionnaire was constructed in line with a MPH master thesis of the Staffordshire University to evaluate for the first time health risks for pathologists in Switzerland. Pressure of work, workplace ergonomics, eye and musculoskeletal problems as well as injuries and allergies constitute main topics of this questionnaire. It will take you about six to ten minutes time to answer all questions. The evaluation will be done completely anonymous in Zurich. Results of this study are to be published in a biomedical journal. In any case you will be informed about the results via the SSP. For any further questions please do not hesitate to contact Florian.Fritzsche@usz.ch (Phone: 044 255 3311) any time. As a little gratitude for your participation MoMoll has sponsored an ergonomic chair (Capisco Puls, see image) that will be raffled off among all returned answers (please tick box below).
To take part in the study please enter your name and address in the field below and tick the respective box to confirm your consent with the study. The link to the questionnaire will appear directly after you have pushed the button "Send the questionnaire" at the end of this consent form. Any backtracking of the sent questionnaire is technically impossible.

Thank you very much for your collaboration!

| **1** | **Please enter here your name, surname and contact data: (compulsory question)** |
| --- | --- |
|  |

| **2** | **The evaluation of the linked questionnaire “Occupational health risks of pathologists in Switzerland” will be completely anonymous. My participation in this study is voluntary. Since after the electronic submission of the questionnaire backtracking to a person is impossible, a later selective exclusion of data is not possible. There is no known risk or harm by participating in this study. My separately sent personal data (name/address) will only be used for the raffle and will not be given to third parties with the exception of lawful obligations. All data will be securely stored in Zurich and will be securely deleted after 10 years. I was informed in written form about aims and course of the study and I have understood this information. (compulsory question)** |
| --- | --- |
|  |

|  |  | I have read this information and declare my informed consent to take part in this questionnaire-based study |
| --- | --- | --- |

| **3** | **Participate in the raffle:  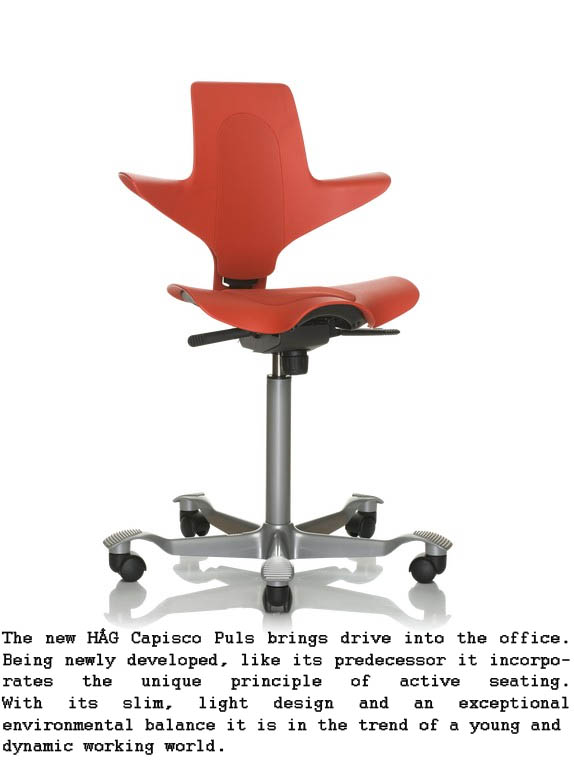** |
| --- | --- |
|  |

|  |  | Yes, I would like to take part in the raffle. My name and address data will only be used for the delivery of the chair if I win. |
| --- | --- | --- |

# Thank you very much for your collaboration! Please click the link below to get to the anonymous questionnaire. to the questionnaire

#

# Occupational Health Risks of Pathologists in Switzerland

Dear pathologist,

thank you very much for taking your time to answer this anonymous questionnaire. Most questions require only one answer. Questions where multiple answers are possible are respectively labelled.

| **1** | **Gender** |
| --- | --- |
|  |

|  |  | Woman |
| --- | --- | --- |
|  |  | Man |

| **2** | **Age** |
| --- | --- |
|  |

|  |  | 25-35 years |
| --- | --- | --- |
|  |  | 36-45 years |
|  |  | 46-55 years |
|  |  | >55 years |

| **3** | **Please describe your position in pathology.** |
| --- | --- |
|  |

|  |  | I am a resident |
| --- | --- | --- |
|  |  | I am a consultant |

|  |  | For how many years have you been working in this position? |
| --- | --- | --- |

| **4** | **Where do you work?** |
| --- | --- |
|  |

|  |  | Private practice |
| --- | --- | --- |
|  |  | University hospital |
|  |  | Non-University hospital |

| **5** | **Are you employed or self-employed?** |
| --- | --- |
|  |

|  |  | Employed |
| --- | --- | --- |
|  |  | Self-employed |

| **6** | **What are your mean weekly hours of work?** |
| --- | --- |
|  |

|  |  | ≤50 h |
| --- | --- | --- |
|  |  | >50 h |
|  |  | >60 h |

| **7** | **Do you work part-time?** |
| --- | --- |
|  |

|  |  | No |
| --- | --- | --- |
|  |  | Yes - ≥80% |
|  |  | Yes - ≥60% |
|  |  | Yes - <60% |

|  |  | Part-time work is only for a short-term period |
| --- | --- | --- |
|  |  | I work part-time for >3 years |

| **8** | **Is your work good manageable in regular working time?** |
| --- | --- |
|  |

|  |  | Yes |
| --- | --- | --- |
|  |  | No |

| **9** | **I the workflow predominantly organised efficiently?** |
| --- | --- |
|  |

|  |  | Yes |
| --- | --- | --- |
|  |  | No |

| **10** | **Are research/teaching/administration relevant parts of your working time?** |
| --- | --- |
|  |

|  |  | Yes - about how many percent: |
| --- | --- | --- |
|  |  | No |

| **11** | **Do you work at the microscope daily?** |
| --- | --- |
|  |

|  |  | Yes - about how many hours daily: |
| --- | --- | --- |
|  |  | No |

| **12** | **Do you work at the computer daily?** |
| --- | --- |
|  |

|  |  | Yes - about how many hours daily: |
| --- | --- | --- |
|  |  | No |

| **13** | **How is the working atmosphere in your team?** |
| --- | --- |
|  |

|  |  | Very good |
| --- | --- | --- |
|  |  | Good |
|  |  | Medium |
|  |  | Bad |

| **14** | **My office...** |
| --- | --- |
|  |

|  | **Yes** | **No** |
| --- | --- | --- |
| has at least 1 window |  |  |
| is air-conditioned |  |  |
| is shared with other colleagues |  |  |

| **15** | **My microscope...** |
| --- | --- |
|  |

|  | **Yes** | **No** |
| --- | --- | --- |
| has a tube adjustable in height |  |  |
| has a condenser lens |  |  |
| is regularly serviced |  |  |
| allows a horizontal line of sight 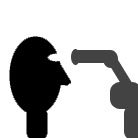 |  |  |

|  |  | is manufactured by (e.g. Zeiss Axioscope 40): |
| --- | --- | --- |

| **16** | **My office chair...** |
| --- | --- |
|  |

|  | **Yes** | **No** |
| --- | --- | --- |
| is individually adjustable (height/back rest) |  |  |
| is ergonomically optimised |  |  |

| **17** | **My desk...** |
| --- | --- |
|  |

|  | **Yes** | **No** |
| --- | --- | --- |
| is sufficiently adjustable in height |  |  |
| is big enough |  |  |
| is adjustable in inclination |  |  |

| **18** | **Have you ever had an introduction into workplace ergonomics and correct sitting positions?** |
| --- | --- |
|  |

|  |  | Yes - last time how many years ago: |
| --- | --- | --- |
|  |  | No |

| **19** | **Have you ever had work-related musculoskeletal problems?** |
| --- | --- |
|  |

|  |  | Yes |
| --- | --- | --- |
|  |  | No |

| **20** | **If you have answered the last question with "Yes", where were these problems localised?** |
| --- | --- |
|  |

(Multiple answers are possible)

|  |  | Neck |
| --- | --- | --- |
|  |  | Shoulder |
|  |  | Upper back |
|  |  | Lower back |
|  |  | Arm/hand |
|  |  | Other locations: |

| **21** | **Did you suffer during the last 4 weeks from such problems?** |
| --- | --- |
|  |

|  |  | Yes |
| --- | --- | --- |
|  |  | No |

| **22** | **Have you been signed off sick due to musculoskeletal problems during the last year?** |
| --- | --- |
|  |

|  |  | Yes - for about how many days: |
| --- | --- | --- |
|  |  | No |

| **23** | **Do you do short breaks regularly for stretching exercises?** |
| --- | --- |
|  |

|  |  | Yes |
| --- | --- | --- |
|  |  | No |

| **24** | **If you use an ergonomically optimised chairs or microscopes, did you experience any effects from this on your musculoskeletal problems?** |
| --- | --- |
|  |

|  |  | I have always used ergonomically optimised chairs/microscopes - no comparison possible |
| --- | --- | --- |
|  |  | I never had any such problems, neither before nor after I started using ergonomically optimised chairs/microscopes |

|  | **declined** | **stayed the same** | **become worse** |
| --- | --- | --- | --- |
| With the use of an ergonomically optimised microscope my problems have... |  |  |  |
| With the use of an ergonomically optimised chair my problems have... |  |  |  |

| **25** | **Do you have an ametropia?** |
| --- | --- |
|  |

|  |  | Yes - myopia |
| --- | --- | --- |
|  |  | Yes - hyperopia |
|  |  | Yes - other or combination: |
|  |  | No |

| **26** | **Did the ametropia (if present) already exist before you started working in pathology?** |
| --- | --- |
|  |

|  |  | Yes |
| --- | --- | --- |
|  |  | No |

| **27** | **Has there been an aggravation of your ametropia (if present) since you started working in pathology?** |
| --- | --- |
|  |

|  |  | Yes - about how many dioptres: |
| --- | --- | --- |
|  |  | No |

| **28** | **Did you suffer from eye fatigue (symptoms: prickle, irritation, dry eyes, blurred vision, headache, malaise) within the last 4 weeks?** |
| --- | --- |
|  |

|  |  | Yes |
| --- | --- | --- |
|  |  | No |

| **29** | **Have you ever been injured working in pathology?** |
| --- | --- |
|  |

|  |  | Yes - doing what (e.g. dissection or autopsy): |
| --- | --- | --- |
|  |  | No |

| **30** | **What kind of injuries did you experience?** |
| --- | --- |
|  |

(Multiple answers are possible)

|  |  | Needle stitch |
| --- | --- | --- |
|  |  | Cutting injury |
|  |  | Splash onto mucosal membranes |
|  |  | Other: |

| **31** | **Did any of these injuries occur during the last year?** |
| --- | --- |
|  |

|  |  | Yes |
| --- | --- | --- |
|  |  | No |

| **32** | **Did any permanent damages result from the injuries?** |
| --- | --- |
|  |

|  |  | Yes |
| --- | --- | --- |
|  |  | No |

| **33** | **Did you ever experience intolerance reactions against formaldehyde (e.g. severe mucosal or skin irritation, mucosal inflammation, fatigue, sleep disorders)?** |
| --- | --- |
|  |

|  |  | Yes |
| --- | --- | --- |
|  |  | No |

| **34** | **Do you have any known allergies?** |
| --- | --- |
|  |

|  |  | Yes - against: |
| --- | --- | --- |
|  |  | No |

| **35** | **Did this/these allergy/ies already exist before you started working in pathology?** |
| --- | --- |
|  |

|  |  | Yes |
| --- | --- | --- |
|  |  | No |

| **36** | **Since working in pathology, did you ever come down with...?** |
| --- | --- |
|  |

|  | **Yes** | **No** |
| --- | --- | --- |
| Tuberculosis |  |  |
| Positive Tuberculin test only |  |  |
| Hepatitis B |  |  |
| Hepatitis C |  |  |
| HIV |  |  |
| Burnout |  |  |
| Depression (except during or following pregnancy) |  |  |
| Hypertension (except during pregnancy) |  |  |
| Diabetes mellitus type II (except during pregnancy) |  |  |
| Malignancies - subtype (e.g. epithelial/haematological/mesenchymal) : |  |  |

| **37** | **Are you sufficiently immunised against hepatitis B?** |
| --- | --- |
|  |

|  |  | Yes |
| --- | --- | --- |
|  |  | No |

| **38** | **Have you ever received a BCG immunisation (tuberculosis)?** |
| --- | --- |
|  |

|  |  | Yes |
| --- | --- | --- |
|  |  | No |

| **39** | **Do you regularly dissect organs or do autopsies?** |
| --- | --- |
|  |

|  |  | Yes |
| --- | --- | --- |
|  |  | No |

| **40** | **Are there cut-resistant gloves available?** |
| --- | --- |
|  |

|  |  | Yes |
| --- | --- | --- |
|  |  | No |

| **41** | **Do you use cut-resistant gloves regularly for dissection or autopsies?** |
| --- | --- |
|  |

|  |  | Yes |
| --- | --- | --- |
|  |  | No |

| **42** | **Do you consider the extraction of air in the dissection room sufficient?** |
| --- | --- |
|  |

|  |  | Yes |
| --- | --- | --- |
|  |  | No |

| **43** | **Do you smoke?** |
| --- | --- |
|  |

|  |  | Yes - how many pack years: |
| --- | --- | --- |
|  |  | No |

| **44** | **Do you go in for sports?** |
| --- | --- |
|  |

|  |  | Yes |
| --- | --- | --- |
|  |  | No |

| **45** | **If you go in for sports, please specify frequency and disciplines.** |
| --- | --- |
|  |

|  | |  | >1x/week | | --- | --- | |  | |  | 1x/week | | --- | --- | |  | |  | >1x/month | | --- | --- | |  | |  | <1x/month | | --- | --- | |
| --- | --- | --- | --- | --- | --- | --- | --- | --- | --- | --- | --- | --- | --- | --- | --- |

(Multiple answers are possible)

|  |  | Endurance sport |
| --- | --- | --- |
|  |  | Muscle sport |
|  |  | Team sport or others |

| **46** | **How would you rate your work-related situation in terms of the next...?** |
| --- | --- |
|  |

|  | **Very good** | **Good** | **Rather bad** | **Bad** |
| --- | --- | --- | --- | --- |
| 2 years |  |  |  |  |
| 5 years |  |  |  |  |

| **47** | **How would you rate the medical relevance of the discipline of pathology in terms of the next 5-10 years?** |
| --- | --- |
|  |

|  | |  | Decreasing | | --- | --- | |  | |  | Remaining the same | | --- | --- | |  | |  | Increasing | | --- | --- | |
| --- | --- | --- | --- | --- | --- | --- | --- | --- | --- | --- | --- |

|  |  | Why? |
| --- | --- | --- |

| **48** | **Please enter here any further comments or ideas. THANK YOU VERY MUCH!** |
| --- | --- |
|  |

**Thank you very much for your participation! You can close the internet-browser now. For any further questions please contact the email address listed below.**
